# Use of Objective Outcomes Measures to Verify the Effects of ICF-Based Gait Treatment in Huntington's Disease Patient on Globus Pallidus Deep Brain Stimulation: A Case Report

**DOI:** 10.3389/fresc.2022.849333

**Published:** 2022-04-14

**Authors:** Tamine T. C. Capato, Rubens G. Cury, Juliana Tornai, Erich T. Fonoff, Renata Guimarães, Manoel T. Jacobsen, Mônica S. Haddad, Egberto R. Barbosa

**Affiliations:** ^1^Department of Neurology, Movement Disorders Center, University of São Paulo, São Paulo, Brazil; ^2^Radboud University Medical Centre, Nijmegen, Netherlands; ^3^Donders Institute for Brain, Cognition and Behaviour, Nijmegen, Netherlands; ^4^Department of Neurology, Nijmegen, Netherlands; ^5^Centre of Expertise for Parkinson & Movement Disorders, Nijmegen, Netherlands; ^6^PHYSICAL Parkinson's Disease and Movement Disorders Rehabilitation Center, São Paulo, Brazil; ^7^Neurosurgery Division, Department of Neurology, School of Medicine, University of São Paulo, São Paulo, Brazil

**Keywords:** Huntington's disease, rehabilitation, ICF, chorea, physical therapy, deep brain stimulation, gait, multidisciplinary treatment

## Abstract

In advanced stages of in Huntington's disease (HD) gait impairments and severe chorea are usually medication-refractory. The long-term effects on gait in HD of physiotherapy ICF-based management post- globus pallidus deep brain stimulation (GPi DBS) are not well-established. Physiotherapy has been recognized as an essential element in HD treatment. Here, we present a case report of a 56-year-old woman with HD on the advanced stage and severe chorea medication-refractory after GPi-DBS. We performed multidisciplinary motor assessments ICF-based to identify the disability at clinical and home-setting, including environmental and personal factors before and after GPi-DBS surgery and at 11-time points follow-up. The surgery was very successful and directly post GPi-DBS, there were a significant improvement in chorea and a substantial decrease in medication dose. A framework ICF- based physiotherapy protocol with external cues was developed to improve gait was delivered post-surgery and was continued three times/week during 18-months. Physiotherapy sessions consisted of a personalized protocol of exercises with functional movements, balance, and gait training with external cues. Improvements in gait were observed in 3-months post-intervention and were more expressive in 6-months follow-up. Our patient improved substantially HD motor symptoms and her quality of life after GPi-DBS intervention and a physiotherapy program ICF-based. The objective outcomes measures used to assess gait have served as endpoints to assessing the patient's motor profile during the pre-operative period. Assessments were helpful to verify the efficacy of the multidisciplinary intervention in long-term.

**Conclusion:**

Periodically assessing function and disability using outcome improvements may support clinicians' decisions about DBS, medication adjustments and guide physiotherapists to personalize the ICF-based intervention.

## Introduction

Huntington's disease (HD) is a neurodegenerative disease characterized by chorea and cognitive and behavioral impairments (1). Despite pharmacological and surgical treatment, patients with HD continue to present motor impairments (2). When Tetrabenazine is not available, antipsychotics are the most commonly used drugs in HD and are indicated in treating both HD motor and psychiatric symptoms (2, 3). However, in moderate and late stages gait impairments and severe chorea are usually medication-refractory in HD (1, 4, 5). Globus pallidus deep brain stimulation (GPi DBS) has demonstrated improvements in chorea in medication-refractory HD (4, 6, 7). However, HD guidelines provide no multidisciplinary standardized approach supported by assessments and non-pharmacological management after GPi DBS (1, 5, 8–12). Recently, physiotherapy has been recognized as an essential element in HD treatment (8) and physiotherapy interventions have been proposed to improve gait and mobility in individuals with HD (13–16).

A starting point for gait rehabilitation management in HD can be functional assessment, and a model to use is the International Classification of Functioning, Disability, and Health (ICF) (17). However, the long-term effects on gait in HD of physiotherapy ICF-based management post-GPi DBS surgery are not well-established.

## Case Description

This case report describes a 56-year-old woman with a genetically confirmed diagnosed of HD (CARE checklist as a Supplementary File). The patient is in the advanced stage of HD and had severe chorea medication-refractory. ICF-based physiotherapy was used to assess and optimize gait treatment before and after GPi DBS. The patient presented depression, apathy and was treated pharmacologically on an outpatient basis for 13 years (haloperidol 30 mg; olanzapine 25 mg) and treated for 6 years with conventional physiotherapy. Progressively, chorea and other motor symptoms (bradykinesia, postural instability, and dystonia) have worsened and become medication-refractory (Supplementary Video 1). Consequently, the patient has become dependent on a caregiver, due to a significant decrease in functioning and participation in all daily activities.

Aiming to decrease the chorea and improve functional capacity, bilateral GPi DBS was performed. Multidisciplinary ICF-based motor assessments were performed to identify the disability and clinical setting, including environmental and personal factors before and after GPi DBS surgery and at 11 time-points during long-term follow-up (18-months). All motor tests were video-recorded for further analysis. Gait was assessed by Timed up and Go test (TUG) (18); balance was assessed by Berg Balance Scale (BBS) (19), Mini-BESTest (20), and retropulsion test (in Unified Huntington's Disease Rating Scale (UDHRS) (21); functionality was assessed by Functional Independence Measure (FIM) (22); and UDHRS was also used to assess motor severity and functional capacity (21). Supplementary Figure 1 shows Huntington's disease assessment model ICF-based proposed in this case report and outcomes measures used to assess the different domains including codes descriptors and qualifiers.

The surgery was very successful, and directly post GPi DBS (50 Hz) there was a significant improvement in chorea (trunk and limbs) without ameliorating motor performances and a substantial decrease in medication dose was observed (olanzapine 2.5 mg) (Supplementary Video 2). Depression and apathy did not significantly change when antipsychotic medication decreased. Although the involuntary axial movements persisted, they were less pronounced and no significant improvements on UDHRS were found (45 points to 43 points). Therefore, chorea improvement by GPi DBS enabled our patient to restart physical therapy gait interventions that had been interrupted before surgery.

A ICF framework-based physiotherapy protocol with external cues was developed to improve gait post-surgery (Supplementary Material). After 2 weeks hospitalization, patient was discharged home. The physiotherapy protocol was delivered by a physiotherapist specialized in movement disorders 2 weeks later of the patient was discharged home. The physiotherapy protocol was continued three times/week in the long term (18 months). Physiotherapy sessions consisted of a personalized protocol of exercises including functional movements, balance exercises, and gait training with external cues (23–26). All physiotherapy sessions were delivered by a physiotherapist specialized in movement disorders at home (three sessions per week) and also in a rehabilitation clinical setting (one session per week). In total, four 60-min sessions per week were delivered. An overview of the protocol of exercises is described in Supplementary Table 1.

Regular physiotherapy treatment was crucial to improving axial motor symptoms, and functional mobility improvement was observed by the global impression of change. As shown in Supplementary Figure 2 improvements in gait were observed at 3-months post-intervention. After 6-months of follow-up, balance and gait improvements were more pronounced on TUG, BBS, and FIM. Significant improvements were found on UHDRS (scored 38 points). No significant effects were found in the Mini BESTest and retropulsion test. The results were maintained at the 18-month follow-up (Supplementary Figure 2), although the effects had deteriorated slightly over this period. Improvements were retained on UHDRS (38 points). In addition, GPi DBS parameters (180 Hz) were stabilized during this period, as was the medication dose (olanzapine 5 mg) and physical therapy protocol was well adherence and tolerability assessed by interview. No adverse event was reported.

## Discussion

This case report shows that immediately after GPi DBS, the advanced stage HD patient with severe chorea and who was medication-refractory showed substantially improved motor symptoms and quality of life after GPi DBS intervention followed by an ICF-based physiotherapy program. The main findings from this case report are a sustained improvement of chorea after GPi DBS and significantly reduced medication dose, without significant change in depression and apathy. Besides several case reports, few small open-label and retrospective studies outlined the efficacy of haloperidol in the control of abnormal involuntary movements in HD without ameliorating motor performances (2, 3, 6, 7). These studies can corroborate our observations.

The UHDRS includes several scores assessing the primary features of HD (motor, cognitive, behavioral) as well as the overall functional impact of these features; therefore, the bio-psycho-social view can also be accessed by UHDRS, which is as a “standard gold” outcome measure in HD. Furthermore, despite the expected neurodegenerative progression of HD, specialized clinical management after GPi DBS can improve gait impairments, effects which are sustained over the long term (6, 27). Interestingly, at 12-months post-GPi DBS, we observed a sustained improvement in chorea, and the effects of the physiotherapy interventions focused on gait improvement (Supplementary Video 3).

The effects observed on TUG are significant and also higher than the minimal clinical change (2.58 points) proposed by Quinn et al. and the same trends were observed on BBS scores (minimal higher than 5 points) (28).

Despite growing evidence, no pharmacological or non-pharmacological disease-modifying therapy for HD has been identified to date (29). Therefore, multidisciplinary interventions (29), including physiotherapy and aerobic exercises, are promising approaches (8) to improve gait impairments in HD.

Recognition of gait impairments in HD is highly important in clinical practice for many reasons. First, although changes in gait and balance are frequently among the earliest signs of HD (30–33), many patients either do not recognize these signs or have difficulty describing their symptoms. A specific abnormal characteristic of gait and balance may develop early in the HD progression, such as bradykinesia and stride-to-stride variability (30, 32). Often, these characteristics can offer important diagnostic clues before other signs and symptoms become evident (33). Furthermore, disorders of gait and balance cause significant disability, falls, and fall-related injuries in HD (34). Using an ICF-based model, it may be easier to identify the level of patient participation in specific activities and how environmental factors can influence these motor and non-motor aspects. Additionally, the timely recognition of gait and balance disorders is important in clinical management (35), because it can help guide interventions by physicians during the most effective period.

After an initial assessment, we suggest that every patient with HD undergo regular screening for gait impairments using a test battery, which involves a review of the patient's history, a formal physical examination, and a cognitive assessment. The combination of outcomes that will determine the type of intervention and the type of training may involve many parameters (8) and an ICF-based framework.

Regardless of the evidence, the clinical implications and importance of referring DH people after GPi DBS to rehabilitation become evident in this case report. In our clinical experience with HD, gait and balance impairments are often formally assessed; however, formal ICF-based assessments are less frequent. We suspect that many practicing clinicians could further glean important insights from specialized multidisciplinary assessments of gait and balance than is routinely collected at present.

Finally, the ICF-based objective outcomes measures used to assess gait served as endpoints for assessing the patient's motor profile during the pre-operative period. These measures also indicated the optimal time to refer the patient to surgical intervention. Assessments were helpful at verifying the efficacy of the multidisciplinary intervention immediately following the intervention and in the long term. Continuing specialized physiotherapy in high frequency (4 times/week) can help optimize gait improvements in HD in advanced stages after GPi DBS and maintain the results over long periods. The patient and family have considered the long-term treatment helpful, which improved her quality of life. This clinical management strategy can work as a good clinical implication. Work with a biopsychosocial model including formal assessments of the complete multidisciplinary team is important, and it was a limitation in this case report. It would be interesting to include formal speech therapist, occupational therapist, social worker, nurse specialist, and psychologist assessments in future studies.

## Conclusion

Periodical assessments of functioning and disability using outcome improvements may support neuromodulation parameter adjustments, pharmacological adjustments, and support physiotherapists at personalizing ICF-based interventions. Our strategy of continuing specialized physiotherapy interventions over a longer period has good potential for clinical implementation in HD therapy. Further research is needed to corroborate our findings.

## Data Availability Statement

The original contributions presented in the study are included in the article/Supplementary Material, further inquiries can be directed to the corresponding author/s.

## Ethics Statement

The studies involving human participants were reviewed and approved by Comite de Etica e Pesquisa-USP-HOSPITAL DAS CLÍNICAS DA FACULDADE DE MEDICINA DA UNIVERSIDADE DE SÃO PAULO-HCFMUSP. The patients/participants provided their written informed consent to participate in this study. Written informed consent was obtained from the individual(s) for the publication of any potentially identifiable images or data included in this article.

## Author Contributions

TC and RC: research project—conception, organization, and execution, statistical analysis—design, execution, and review and critique, and manuscript—writing of the first draft and review and critique. RG, JT, and MH: research project—execution and statistical analysis—execution. EF: research project—conception and execution and statistical analysis— review and critique. MJ: manuscript—review and critique. EB: research project—execution and manuscript—review and critique. All authors contributed to the article and approved the submitted version.

## Conflict of Interest

The authors declare that the research was conducted in the absence of any commercial or financial relationships that could be construed as a potential conflict of interest.

## Publisher's Note

All claims expressed in this article are solely those of the authors and do not necessarily represent those of their affiliated organizations, or those of the publisher, the editors and the reviewers. Any product that may be evaluated in this article, or claim that may be made by its manufacturer, is not guaranteed or endorsed by the publisher.

## Supplementary Material

Supplementary Figure 1 Huntington's Disease assessment model ICF-based and outcomes measures used to assess the different domains including codes descriptors and qualifiers.

Supplementary Figure 2 Global impression of change of outcomes changes before and after GPI—DBS.

Supplementary Table 1 Overview of physiotherapy intervention contents proposed in this case report.

Supplementary Video 1 Baseline—Before surgery patient presents severe generalized chorea and postural instability which destabilize her. The patient is not able to sit and stand up without assistance.

Supplementary Video 2 Post-intervention—One-month follow-up after surgery, GPi-DBS provides a substantially reduction of chorea, especially of lower limbs.

Supplementary Video 3 Long-term−12-months after surgery. GPi-DBS had provided maintained a reduction of chorea and an improve even more axial symptoms. The patient can walk with walker and unilateral assistance with specialized physicaltherapy interventions. Introduction of aerobic exercises. Facial grimaces are still preset.
