# Supplementary material for: Use of Objective Outcomes Measures to Verify the Effects of ICF-Based Gait Treatment in Huntington's Disease Patient on Globus Pallidus Deep Brain Stimulation: A Case Report
Source: Front Rehabil Sci. 2022 Apr 14;3:849333. doi: 10.3389/fresc.2022.849333 (PMC9397791; doi:10.3389/fresc.2022.849333)
Supplement: Supplementary Figure 1 — Huntington's Disease assessment model ICF-based and outcomes measures used to assess the different domains including codes descriptors and qualifiers. [file Data_Sheet_1.PDF]

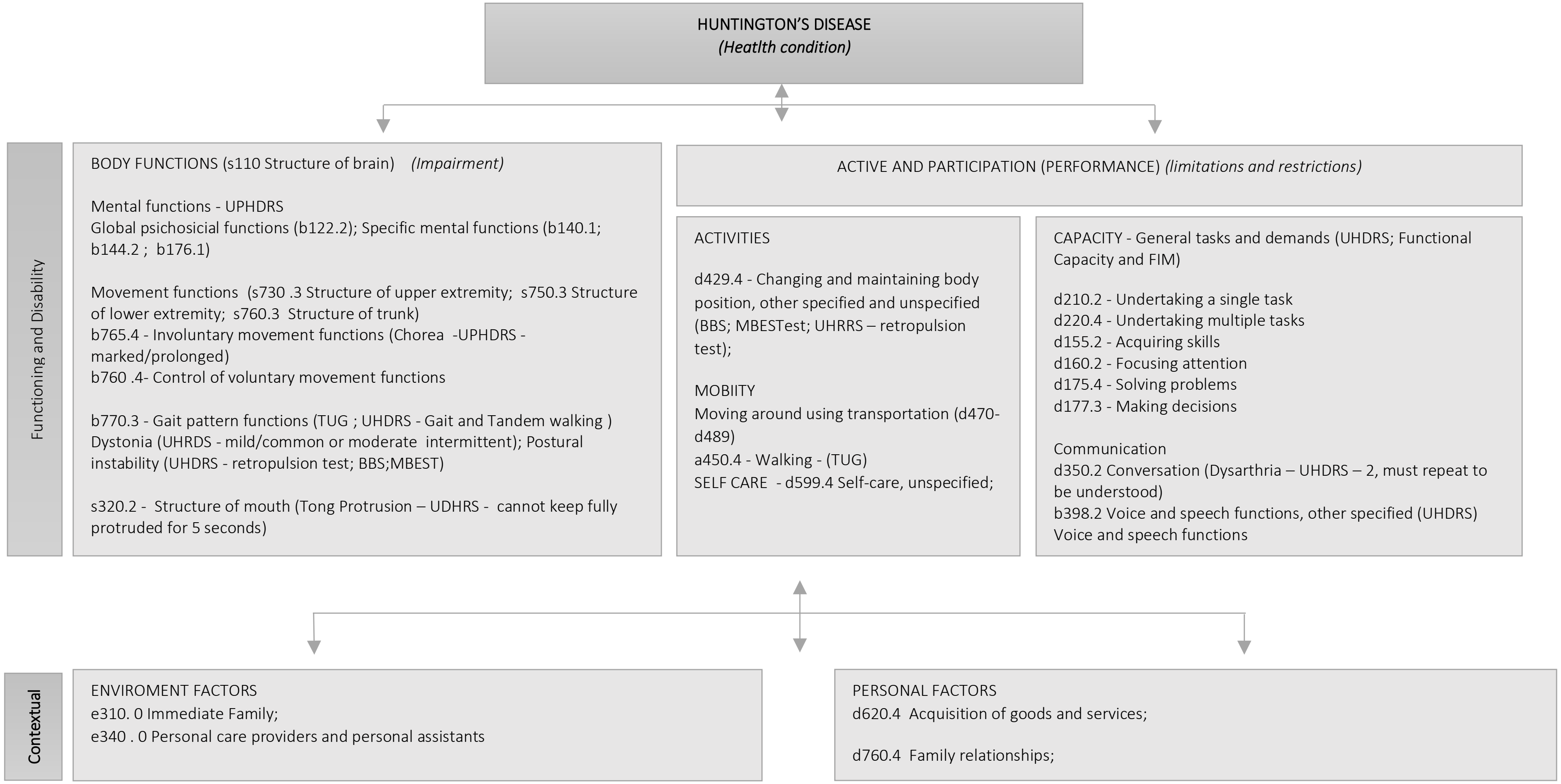

Figure 1: Huntington's Disease assessment model ICF-based and outcomes measures used to assess the different domains including codes descriptors and qualifiers
