# Supplementary material for: Use of Objective Outcomes Measures to Verify the Effects of ICF-Based Gait Treatment in Huntington's Disease Patient on Globus Pallidus Deep Brain Stimulation: A Case Report
Source: Front Rehabil Sci. 2022 Apr 14;3:849333. doi: 10.3389/fresc.2022.849333 (PMC9397791; doi:10.3389/fresc.2022.849333)
Supplement: Supplementary Table 1 — Overview of physiotherapy intervention contents proposed in this case report. [file Data_Sheet_2.PDF]

Supplementary material – Protocol of exercises

Training Sessions	***Intervention*** - Neurological Physiotherapy	***Orientations*** intensity and number of repetition were ajudested once per month according patient improvement
FREQUENCY *4x/week*	**Duration/ Dose/Material** **60 minutes**	**Progression/ Dose**
WARM-UP *10 MINUTES*	Muscle stretching, global movement of upper and lower limbs, and joint movements of wide range. Flexion and extension, lateral inclination and rotation of the trunk; flexion and extension, lateral inclination and rotation of the cervical. Movements were performed with supervision and support.	The exercises were performed lay down and sitting position with the feet on neutral position, together and apart. Decreasing and increasing the base of support. Increasing and decreasing movement velocity and amplitude. (support bar or chair).
MOTOR TRAINING *40 MINUTES*	Divided on 3 parts:<br>1. Functional movements (stand up from the chair, and bend over to pick up different objects (e.g. ball, Sticks) with different weight, size, texture and color).<br>2. Balance - Axial and proximal movements displacements in different planes and axis; coordinated movements with Upper and Lower limbs range and speed; Functional reach, weight shifts in different directions (anterior, posterior and lateral), using foam, using stable ground, mats and disks with textures. Postural reactions, trunk rotation.<br>3. Gait training: stationary and gait training on stable surface using the walker stabilizer. Auditory cues were provided by metronome- 40 bpm, or 60 bpm. Visual cues were provided by straps taped on the floor.	The exercises were performed sitting and stand up position with the basis in neutral position.<br>Progress - not holding on to walker, bar or chair.<br>Progressively increase or decrease the movement speed depending on the exercise (and thereby making the exercise more difficult).<br>Aerobic exercises - stationary bike
COOL DOWN *10 MINUTES*	The execution of exercises (rhythm) becomes progressively slower. Breathing exercises associated to free active movement of upper limbs, global muscular relaxation and stretching. Posture training in up orthostatic position. Cues with metronome 80 BPM	Focusing attention on all sets of exercises: body structure, limbs position, range of motion, posture and basis of support.

Table 1.**Overview of physiotherapy intervention contents proposed in this case report**
