# Supplementary material for: Use of Objective Outcomes Measures to Verify the Effects of ICF-Based Gait Treatment in Huntington's Disease Patient on Globus Pallidus Deep Brain Stimulation: A Case Report
Source: Front Rehabil Sci. 2022 Apr 14;3:849333. doi: 10.3389/fresc.2022.849333 (PMC9397791; doi:10.3389/fresc.2022.849333)
Supplement: Supplementary file 5 [file Data_Sheet_3.PDF]

CARE case report guidelines

CARE Checklist of information to include when writing a case report

(cc) BY-NC-ND

Topic	Item	Checklist item description	Reported on Line
Title	1	The diagnosis or intervention of primary focus followed by the words "case report"	1
Key Words	2	2 to 5 key words that identify diagnoses or interventions in this case report, including "case report"	23
Abstract (no references)	3a	Introduction: What is unique about this case and what does it add to the scientific literature?	29-33
	3b	Main symptoms and/or important clinical findings	33-42
	3c	The main diagnoses, therapeutic interventions, and outcomes	36-54
	3d	Conclusion—What is the main "take-away" lesson(s) from this case?	52-55
Introduction	4	One or two paragraphs summarizing why this case is unique (**may include references**)	78-83
Patient Information	5a	De-identified patient specific information.	83-84
	5b	Primary concerns and symptoms of the patient.	83-90
	5c	Medical, family, and psycho-social history including relevant genetic information	79
	5d	Relevant past interventions with outcomes	83-91
Clinical Findings	6	Describe significant physical examination (PE) and important clinical findings.	92-104 Fig 1 and 2 suplementary material
Timeline	7	Historical and current information from this episode of care organized as a timeline	Figure 2 supplementary material
Diagnostic Assessment	8a	Diagnostic testing (such as PE, laboratory testing, imaging, surveys).	not aplicable
	8b	Diagnostic challenges (such as access to testing, financial, or cultural)	not aplicable
	8c	Diagnosis (including other diagnoses considered)	not aplicable
	8d	Prognosis (such as staging in oncology) where applicable	not aplicable
Therapeutic Intervention	9a	Types of therapeutic intervention (such as pharmacologic, surgical, preventive, self-care)	106-128 Table 1 suplementary ma
	9b	Administration of therapeutic intervention (such as dosage, strength, duration)	118- 1298 and Table 1 suplementary material
	9c	Changes in therapeutic intervention (with rationale)	129- 141;Figure 2 suppmentary material
Follow-up and Outcomes	10a	Clinician and patient-assessed outcomes (if available)	supplement materaila Figure 1 and 2 and videos 1,2,3
	10b	Important follow-up diagnostic and other test results	106- 141; supplement material Figure 1 and 2 and videos 1,2,3
	10c	Intervention adherence and tolerability (How was this assessed?)	140
	10d	Adverse and unanticipated events	140
Discussion	11a	A scientific discussion of the strengths AND limitations associated with this case report	145-166; 200-214
	11b	Discussion of the relevant medical literature **with references**.	144-214
	11c	The scientific rationale for any conclusions (including assessment of possible causes)	172-214
	11d	The primary "take-away" lessons of this case report (without references) in a one paragraph conclusion	216-221
Patient Perspective	12	The patient should share their perspective in one to two paragraphs on the treatment(s) they received	207
Informed Consent	13	Did the patient give informed consent? Please provide if requested	Yes ☒ No ☐
